# Diabetes Alters Contraction-Induced Mitogen Activated Protein Kinase Activation in the Rat Soleus and Plantaris

**DOI:** 10.1155/2008/738101

**Published:** 2008-06-04

**Authors:** A. Katta, D. L. Preston, S. K. Karkala, S. Asano, S. Meduru, S. P. Mupparaju, E. Yokochi, Kevin M. Rice, D. H. Desai, E. R. Blough

**Affiliations:** ^1^Department of Biological Sciences, Marshall University, Huntington, WV 25755, USA; ^2^Department of Exercise Science and Sports Recreation, Marshall University, Huntington, WV 25755, USA; ^3^Departments of Pharmacology, Physiology, and Toxicology, Joan C. Edwards School of Medicine, Marshall University, Huntington, WV 25755, USA; ^4^Cell Differentiation and Development Center, Marshall University, Huntington, WV 25755, USA

## Abstract

The prescription of anaerobic exercise has recently been advocated for the management of diabetes; however exercise-induced signaling in diabetic muscle remains largely unexplored. Evidence from exercise studies in nondiabetics suggests that the extracellular-signal-regulated kinases (Erk1/2), p38, and c-JUN NH2-terminal kinase (Jnk) mitogen-activated protein kinases (MAPKs) are important regulators of muscle adaptation. Here, we compare the basal and the in situ contraction-induced phosphorylation of Erk1/2- p38- and Jnk-MAPK and their downstream targets (p90rsk and MAPKAP-K2) in the plantaris and soleus muscles of normal and obese (fa/fa) Zucker rats. Compared to lean animals, the time course and magnitude of Erk1/2, p90rsk and p38 phosphorylation to a single bout of contractile stimuli were greater in the plantaris of obese animals. Jnk phosphorylation in response to contractile stimuli was muscle-type dependent with greater increases in the plantaris than the soleus. These results suggest that diabetes alters intramuscular signaling processes in response to a contractile stimulus.

## 1. INTRODUCTION

Exercise has long been recognized to have important health benefits for people with type II diabetes. The molecular events underlying exercise-induced adaptations in diabetic muscle remain largely undefined. It is thought that beneficial effects of exercise on structural and functional adaptations of muscle are mediated through the activation of various signaling molecules, which activate signaling cascades involved in regulating changes in gene expression. Similar to that seen with aerobic exercise modalities [1–3], recent data has suggested that anaerobic exercise may also be beneficial in the treatment of diabetes [4–11]. For example, progressive resistance training has been found to improve glycemic control, increase skeletal muscle size and strength, and positively change body composition by increasing lean body mass and decreasing visceral and total body fat [12–15]. Whether diabetes is characterized by differences in how muscle responds to resistance training is not well understood.

Although not fully delineated, it has been postulated that the mitogen activated protein kinase (MAPK) signaling cascade may be particularly important in mediating the effects of altered contractile activity. Indeed, muscle contraction and load-induced muscle hypertrophy in humans and rodents have been found to be associated with the activation of the extracellular signal-regulated kinases 1/2 (Erk1/2), p38, and c-Jun NH_2_-terminal kinase (Jnk) MAPK proteins [16–20]. Several downstream substrates of MAPK signaling have also been identified, including the Erk1/2-dependent protein MAPK-activated proteinkinase-1 (MAPKAP-K1), also called p90 ribosomal S6kinase (p90RSK) [21] and the p38-dependent protein MAPK-activated protein kinase-2 (MAPKAP-K2) [22, 23]. It is thought that the phosphorylation (activation) of MAPK proteins and their substrates is important in regulating such diverse processes as gene expression, glucose uptake, cell replication, and protein synthesis [16, 24, 25]. To our knowledge, the effect of diabetes on contraction-induced MAPK signalingin muscle has not been investigated.

The purpose of this study was to examine whether diabetes alters MAPK signaling after an acute bout of contractile activity. We hypothesized that contraction induced MAPK signaling would differ between normal and diabetic muscles. To test this hypothesis, the phosphorylation status of Erk1/2-, p38-, and Jnk-MAPK and their downstream kinases were analyzed in the plantaris and soleus muscles of the normal and diabetic rats immediately after and during the recovery phase of an acute bout of high-frequency electrical stimulation. Taken together, our data suggests that diabetes alters contraction-induced MAPK phosphorylation in skeletal muscle.

## 2. MATERIALS AND METHODS

### 2.1. Animal care

Young (10 weeks, *n* = 12) male lean normal Zucker (LNZ) and young (10 weeks, *n* = 12) male obese syndrome-X Zucker (OSXZ) rats were obtained from the Charles River Laboratories. Rats were housed to a cage in an AAALAC approved vivarium. Housing conditions consisted of a 12H: 12H dark-light cycle and temperature was maintained at 22° ± 2°*c*. Animals were provided food and water ad libitum and allowed to recover from shipment for at least two weeks before experimentation. During this time, the animals were carefully observed and weighed weekly to ensure none exhibited signs of failure to thrive, such as precipitous weight loss, disinterest in the environment, or unexpected gait alterations. All procedures were performed as outlined in the Guide for the Care and Use of Laboratory Animals as approved by the Council of the American Physiological Society and the institutional Animal Use Review Board.

### 2.2. Materials

Anti-Erk1/2 (#9102), phospho-Erk1/2^Thr202/Tyr204^ (#9106), p38(#9212), phospho-p38^Thr180/Tyr182^ (#9216), SAPK/Jnk (#9252), phospho-SAPK/Jnk^Thr183/Tyr185^ (#9251), p90RSK (#9334), phospho-p90RSK ^Ser380^(#9335), MAPKAP-K2 (#3042), phospho-MAPKAP-K2^Thr334^ (#3007), Mouse IgG, and Rabbit IgG antibodies were purchased from Cell Signaling Technology (Beverly, Mass, USA). Enhanced chemiluminescence (ECL) western blotting detection reagent was from Amersham Biosciences (Piscataway, NJ, USA). Restore western blot stripping buffer was obtained from Pierce (Rockford, Ill, USA), and 3T3 cell lysates were from Santa Cruz Biotechnology (Santa Cruz, Calif, USA). Antirabbit and antimouse fluorescein antibodies and VectaSheild Hardset Mounting Medium with DAPI was purchased from Vector Laboratories (Burlingame, Calif, USA). All other chemicals were purchased from Sigma (St. Louis, Mo, USA) or Fisher Scientific (Hanover, Ill, USA).

### 2.3. Contractile stimulation of skeletal muscles

The high-frequency electrical stimulation (HFES) model has been previously described [26] and was chosen on the basis of its efficacy in stimulating protein translation and muscle hypertrophy in vivo [27]. By directly stimulating the motor nerve, this model elicits a maximal contraction that when repeated over time produces a muscle adaptation that closely mimics that seen in humans following a program of resistance training. HFES loading of the rat hindlimb was performed using a voltage of 5–7 V (1-millisecond pulses, 100 Hz) as described previously [27]. These conditions have been shown to produce a maximal contraction. Muscle contractions lasted 3 seconds and were followed by a 10-second rest. Ten sets of 6 repetitions were performed with each set being separated by a 50-second rest period. This protocol results in concentric (shortening) contraction of the plantaris and soleus. The fast-twitch plantaris (PLA) and slow-twitch soleus (Sol) muscles were chosen on the basis of previous studies demonstrating that HFES induces MAPK phosphorylation in these muscles [17, 28–30]. Animals were killed by drug overdose at baseline, immediately following, 1hour or 3 hours (*n* = 6 normal, *n* = 6 diabetic for 0, 1, and 3 hours) after HFES. Once excised, muscles were blotted dry, trimmed of visible fat and tendon projections, weighed, immediately frozen in liquid nitrogen, and stored at −80°C.

### 2.4. Preparation of protein isolates and immunoblotting

Muscles were pulverized in liquid nitrogen using a mortar and pestle until a fine powder was obtained. After washing with ice cold PBS, pellets were lysed on ice in for 15 minutes in T-PER (2 mL/1 g tissue weight) (Pierce, Rockford, Ill, USA) and centrifuged for 10 minutes at 2000 X g to pellet particulate matter. This process was repeated twice and the supernants combined for protein concentration determination using the Bradford method (Pierce, Rockford, Ill, USA). Samples were diluted to a concentration of 3 *μ*g/*μ*L in SDS loading buffer, boiled for 5 minutes, and 60 *μ*g of protein were separated using 10% SDS-PAGE gels. Transfer of protein onto nitrocellulose membranes, verification of transfer, and determination of equal loading between lanes and membranes was determined as outlined previously [31]. Protein immunodetection was performed as outlined by the antibody manufacturer while immunoreactive bands were visualized with ECL (Amersham Biosciences, NJ, USA). Exposure time was adjusted to keep the integrated optical densities within a linear and nonsaturated range. Band signal intensity was normalized to *β*-actin by densitometry using a flatbed scanner (Epson Perfection 3200 PHOTO) and imaging software (AlphaEaseFC). Molecular weight markers (Cell Signaling) were used as molecular mass standards, and NIH 3T3 cell lysates were included as positive controls. To allow direct comparisons to be made between the concentration levels of different signaling molecules, immunoblots were stripped and re-probed with Restore western blot stripping buffer as detailed by the manufacturer (Pierce, Rockford, Ill, USA).

### 2.5. Statistical analysis

Results are presented as *mean* ± *SEM*. Data were analyzed by using the Sigma Stat 3.0 statistical program. Effects of exercise on type II diabetes mellitus in the presence of metabolic syndrome were analyzed using a two-way ANOVA followed by the Student-Newman-Keuls post hoc testing when appropriate. A *P* value < .05 was considered to be statistically significant.

## 3. RESULTS

Average body mass of obese Zucker rats was ∼82% higher than lean counterparts (*P* < .05) (see Table 1). Plantaris and solues muscle mass in obese Zuckers, as a percentage of body weight, was only ∼34% and ∼58%, respectively, of muscle mass in lean Zuckers (*P* < .05) (see Table 1).

### 3.1. MAPK protein levels and phosphorylation status are altered in the diabetic soleus and plantaris

**Figure 1:**
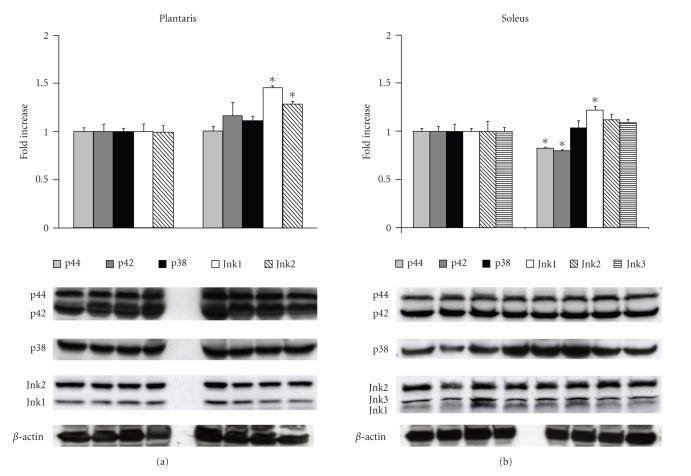
Type 2 diabetes is associated with alterations in skeletal muscle MAPK proteins. Plantaris and soleus muscles from LNZ and obese (fa/fa) Zuckers (OSXZ) were analyzed by Western blot analysis for diabetes-related changes in total Erk1/2-, p38 MAPK, and Jnk protein expression. Protein quantification was normalization to the abundance of *β*-actin. Results are expressed as a percent of the normal, LNZ value. An asterisk (*) indicates significant differences (*P* < .05) from the lean Zucker value.

Immunoblotting analysis of proteins normalized to *β*-actin indicated that there were no significant differences in total p38 MAPK content in plantaris and solues in the OSXZ compared to the LNZ (*P* < .05) (see Figure 1). Plantaris Erk1/2 (p44/p42) content was not significantly different between the groups, while Erk1/2(p44/p42) content in the OSXZ soleus was 17.0 ± 4.8% and 20.3 ± 6.9% lower (*P* < .05) than that observed in their lean counterparts (see Figure 1). Jnk1 levels were 46.1 ± 10.2% higher in OSXZ plantaris and 22.2 ± 7.2% higher in the OSXZ soleus, compared to LNZ (*P* < .05) (see Figure 1). Jnk2 expression was 29.0 ± 9.34% higher in OSXZ plantaris as compared to lean counterparts (*P* < .05), with no significant difference in the OSXZ soleus muscle (see Figure 1). There was no detectable expression of Jnk3 in the plantaris, while Jnk3 content did not differ between LNZ and OSXZ solei. There were no significant differences between OSXZ and LNZ in p90RSK, or MAPKAP-K2 protein levels for either the plantaris or soleus (data not shown).

**Figure 2:**
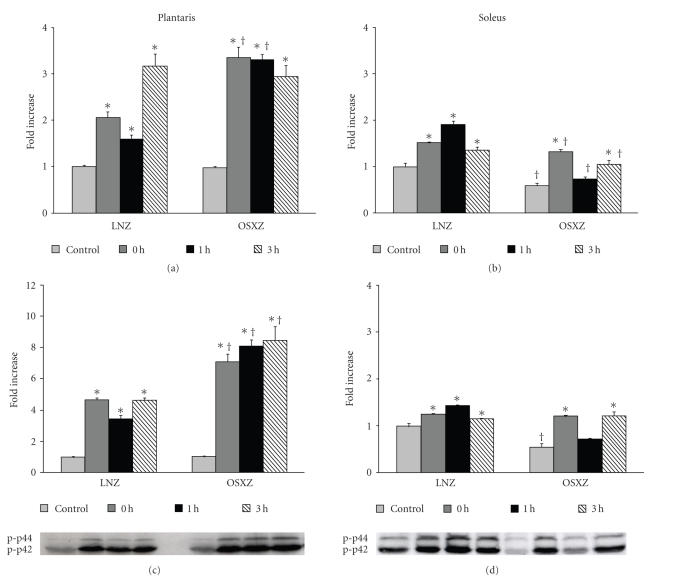
Contraction-induced Erk1/2-MAPK phosphorylation is altered with type II diabetes. Plantaris and soleus muscles from lean (LNZ) and obese Zucker (OSXZ) rats were obtained immediately after, 1 and 3 hours after a bout of HFES. Alterations in Erk1 (a),(b) and Erk2 (c),(d)-MAPK phosphorylation with exercise were determined by immunoblotting. An asterisk (*) indicates significant difference (*P* < .05) from the control within animal model, and a cross (^†^) indicates significant difference (*P* < .05) at corresponding time points across animal models.

**Figure 3:**
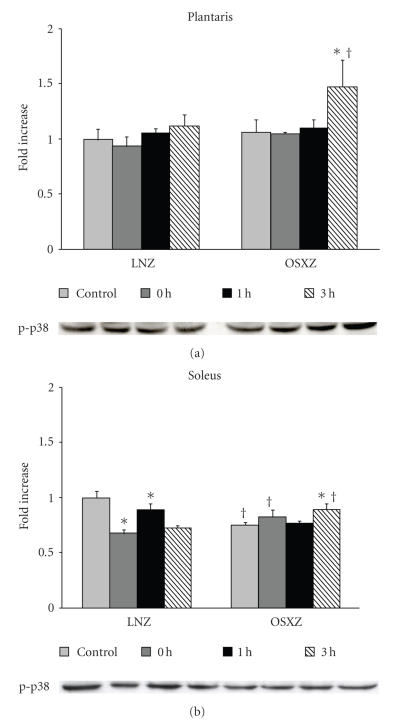
Effects of concentric, maximal muscle contraction in situ on phosphorylation of p38-MAPK. The basal (control) and contraction-induced phosphorylation of the Jnk in plantaris and soleus muscles from lean (LNZ) and obese Zucker (OSXZ) rats at 0, 1, and 3 hours after HFES. Phosphorylation of p38-MAPK was determined by immunodetection of phosphorylation on Thr180 and Tyr182 (phospho-p38 *α* MAPK). An asterisk (*) indicates significant difference (*P* < .05) from the control time point within animal model, and a cross (^†^) indicates significant difference (*P* < .05) at corresponding time points across animal models.

**Figure 4:**
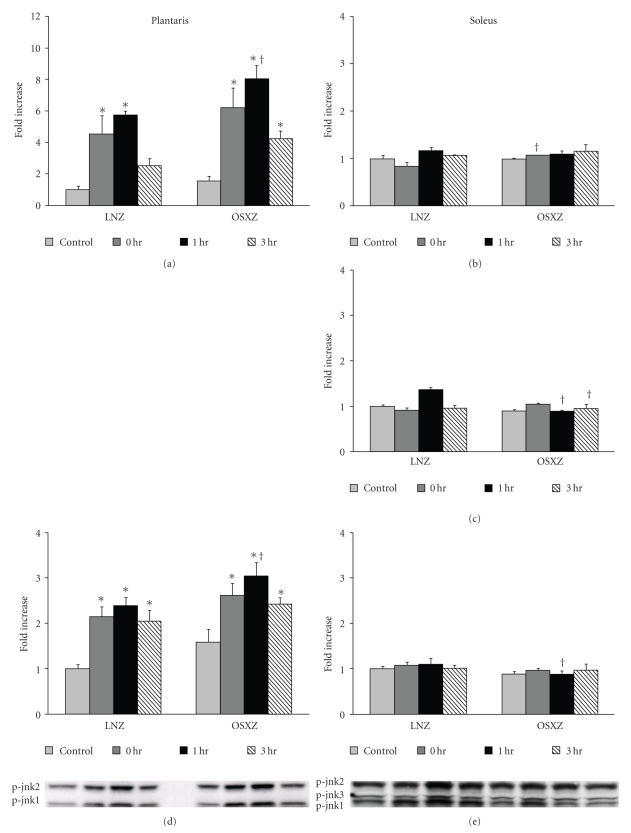
Effects of concentric, maximal muscle contraction in situon phosphorylation of Jnk. The basal (control) and contraction-induced phosphorylation of the Jnk1 (d),(e), Jnk2 (a),(b), Jnk3-MAPK (c) in plantaris and soleus muscles from lean and diabetic Zucker rats at 0, 1, and 3 hours after contractile stimulus. Jnk phosphorylation was determined by Western analysis and immunodetection for Jnk phosphorylation on Thr183 and Tyr185. An asterisk (*) indicates significant difference (*P* < .05) from the control within animal model, and a cross (^†^) indicates significant difference (*P* < .05) at corresponding time points across animal models.

**Figure 5:**
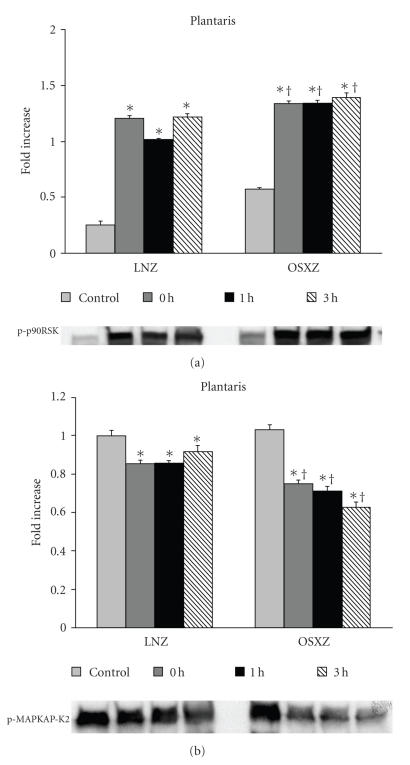
Contraction-induced p90RSK (MAPKAP-K1) and MAPKAPK-2 phosphorylation are altered with type II diabetes. Plantaris muscles from lean (LNZ) and obese Zucker (OSXZ) rats were obtained immediately after, 1 and 3 hours after a bout of HFES. Alterations in p90RSK (a) and MAPKAPK-2 (b) phosphorylation with exercise were determined by immunoblotting. An asterisk (*) indicates significant difference (*P* < .05) from the control within animal model, and a cross (^†^) indicates significant difference (*P* < .05) at corresponding time points across animal models.

Basal levels of phosphorylated forms of MAPK proteins were measured to determine if diabetes altered MAPK activation. Immunoblot analysis using phospho-specific antibodies indicated that the basal level of phospho-Erk1/2 was 41.2 ± 13.3% and 45.1 ± 13.4% lower (*P* < .05), respectively, in OSXZ soleus compared to LNZ, with no significant difference in the plantaris (see Figure 2). The basal level of p38 phosphorylation was unchanged in the plantaris and was 24.9 ± 8.7% lower in the OSXZ versus the LNZ soleus (*P* < .05) (see Figure 3). There was no difference in the basal expression of any of the phosphorylated Jnk isoforms between LNZ and OSXZ muscles (see Figure 4). In the plantaris, the basal phosphorylation level of p90s6k was 128.3 ± 19.28% higher OSXZ compared to the LNZ rats (*P* < .05) while the basal phosphorylation of MAPKAP-K2 was unchanged (see Figure 5).

### 3.2. MAPK phosphorylation in response to contractile stimulus is altered by diabetes

Phosphorylation of p38, Erk1/2, and Jnk-MAPK was determined at 0, 1, and 3 hours after a bout of HFES for comparison with control values. In the case of each molecule examined, significant differences existed between lean and obese Zucker rat models. Differences also were present between the predominately fast twitch plantaris and slow twitch soleus (see Figures 2–4). LNZ plantaris muscles showed no differences in p38 phosphorylation status post exercise. Conversely, the OSXZ plantaris exhibited a 38.5 ± 33.8% increase in phosphorylation from baseline at 3-hours post exercise (*P* < .05) (see Figure 3). The LNZ soleus had a 33.5 ± 9.7% and 27.2 ± 8.0% decrease in phosphorylation of p38 at 1 and 3 hours post exercise, respectively, while OSXZ soleus muscles showed an increase of 18.8 ± 10.7% at 3 hours post exercise from the baseline (*P* < .05) (see Figure 3).

Both Erk1/2 MAP kinases showed significant increases in phosphorylation levels from baseline in response to the contractile stimulus. In the plantaris muscle, LNZ animals had an increase in phosphorylation of Erk1 (p44) of 106.1 ± 13.8%, 59.8 ± 10.9%, and 215.9 ± 29.5% at 0, 1, and 3hours, respectively. OSXZ animals showed an increase of 244.3 ± 24.7%, 239.3 ± 13.9%, and 200.3 ± 28.7% at 0, 1, and 3 hours, respectively, (*P* < .05) (see Figure 2). The slow twitch soleus muscle of LNZ had an increase in the phosphorylation level of p44 by 51.3 ± 9.3%, 90.3 ± 14.9%, and 36.8 ± 13.7% from baseline at 0, 1, and 3 hours, respectively, (*P* < .05). The soleus muscles of OSXZ animals responded differently with increases in p44 phosphorylation of 124.7 ± 19.7% and 78.5 ± 25.9% at 0 and 3 hours, respectively, (*P* < .05) (see Figure 2). In LNZ animals, the plantaris exhibited increases of Erk2 (p-42) phosphorylation of 368.5 ± 16.6%, 244.2 ± 29.8%, and 364.1 ± 22.3%, while contraction in OSXZ elicited increases in p-42 phosphorylation of 582.7 ± 55.2%, 679.0 ± 48.2%, and 716.6 ± 9.2% at 0, 1, and 3 hours, respectively, (*P* < .05) (see Figure 2). LNZ soleus muscles increased p42 phosphorylation by 24.8 ± 55.2%, 43.3 ± 55.2%, and 15.5 ± 55.2% above baseline at 0, 1, and 3 hours, respectively, (*P* < .05). This response appeared greater in OSXZ solei as soleus contraction in these animals increased the phosphorylation of p42 by 120.9 ± 55.2%, 30.5 ± 55.2%, and 122.0 ± 55.2% at 0, 1, and 3 hours, respectively, (*P* < .05) (see Figure 2).

Similar to ERK1/2 and p38, muscle contraction also increased the phosphorylation of Jnk isoforms. In the LNZ plantaris, Jnk1 phosphorylation increased 115.2 ± 31.9%, 140.0 ± 27.9%, and 105.4 ± 34.5%, and Jnk2 by 354.2 ± 140.3%, 472.8 ± 49.7%, and 153.0 ± 70.0% from baseline at 0, 1, and 3 hours, respectively, (*P* < .05). In obese animals, Jnk1 phosphorylation with contraction was increased by 65.2 ± 35.3%, 92.7 ± 36.5%, and 52.7 ± 27.1%, and Jnk2 by 300.4 ± 97.8%, 417.8 ± 72.2% and 174.5 ± 58.2% from baseline at 0, 1, and 3 hours, respectively, (*P* < .05) (see Figure 4). In LNZ soleus muscles, HFES did not change the phosphorylation level of Jnk1 and Jnk2 from that seen in control animals, however the phosphorylation level of Jnk3 increased 36.7 ± 7.2% from baseline immediately after exercise (*P* < .05). HFES did not alter Jnk1, Jnk2, or Jnk3 phosphorylation in the OSXZ soleus (see Figure 4).

To determine whether differences in MAPK signaling with type II diabetes were associated with changes in the regulation of potential downstream MAPK targets, we examined the regulation of p90RSK and MAPKKAP-K2. Contraction induced significant increases in phosphorylation levels of p90s6k (downstream target of Erk1/2) from baseline in the plantaris muscles of both normal and diabetic animals. In the LNZ animals, HFES increased the phosphorylation level of p90s6k by 382.2 ± 22.3%, 304.4 ± 17.4%, and 387.8 ± 24.7% from baseline at 0, 1, and 3 hours, respectively. In a similar fashion, plantaris muscles from obese animals demonstrated increases in phosphorylation of p90s6k of 134.0 ± 7.6%, 135.5 ± 6.7%, and 144.5 ± 9.5% from baseline at 0, 1, and 3 hours, respectively (*P* < .05) (see Figure 5). Conversely, the plantaris muscles of LNZ had a decrease in phosphorylation level of MAPKAP-K2 (downstream target of p38) by 14.5 ± 4.6%, 14.3 ± 4.1%, and 8.1 ± 6.0% from baseline at 0, 1, and 3 hours, respectively. Plantaris muscles of OSXZ animals responded in a similar way and exhibited decreases in MAPKAP-K2 phosphorylation of 27.5 ± 4.2%, 31.1 ± 4.6%, and 40.0 ± 5.6% at 0, 1, and 3 hours, respectively, (*P* < .05) (see Figure 5). No evidence of p90RSK or MAPKAP-K2 phosphorylation in any of the soleus muscles examined (data not shown).

## 4. DISCUSSION

 The obese Zucker (*fa/fa*) rat is an animal model of severe skeletal muscle insulin resistance that is also characterized bymarked hyperinsulinemia and glucose intolerance, dyslipidemia, and central adiposity and therefore is a suitable animal model of the insulin resistance syndrome [32]. It is thought that the mechanisms underlying the progression of insulin resistance in these animals closely mimic that seen in human type II diabetes [33]. The objective of this study was to examine how type II diabetes affects how skeletal muscle responds to a contractile stimulus. Our findings suggest that diabetes is associated with alterations in how muscle contraction regulates MAPK signaling and are consistent with the notion that diabetes may affect the regulation of these proteins differently depending upon fiber type.

## 5. TYPE II DIABETES ASSOCIATED ALTERATION IN MAPK PROTEIN CONTENT AND BASAL PHOSPHORYLATION LEVELS

In mammalian cells, the Erk1/2-, p38-, and Jnk-MAPK pathways are thought to be the three major signaling cascades comprising the MAPK signaling system [34]. When compared to lean animals, the diabetic plantaris and soleus muscles exhibited increased amounts of Jnk-1 while in the diabetic soleus, the amount of Erk1/2 and phosphorylated Erk1/2 was diminished (see Figures 1 and 2). Previous studies examining Jnk levels in human skeletal muscle [35] and Erk1/2 signaling in the obese Zucker gastrocnemius [36] have yielded similar results. It is unclear why diabetes may induce differences in the way different muscle types might regulate the amount and basal activation of these proteins. Indeed, previous studies investigating the effect of increased glucose or the influence of diabetes on MAPK protein levels and phosphorylation state have been equivocal. For example, some studies have demonstrated no effect [37] while others have suggested that altered glucose levels can increase [38] or diminish the phoshorylation of these proteins [39]. The factor(s) responsible for differences between studies are unknown but could be related to cell type-specific responsesor to differences in the concentrations of glucose tested or in the severity of hyperglycemia observed in the experimental preparation. Similarly, diabetes-associated changes in muscle fiber type may also be involved. For example, Punkt and colleagues have recently demonstrated that slow oxidative fiber fraction was reduced by 16% with diabetes while the fast glycolytic fiber fraction was increased by 49% in vastus lateralis of type II diabetic patients [40]. Given that the regulation of MAPK proteins may differ between fiber types [41, 42], it is possible that fiber type transitions associated with diabetes may be involved. Finally, recent data has suggested that fast- and slow-twitch muscles may rely on different signaling mechanisms to mediate glucose uptake [43, 44]. Whether these differences explain some or all of the alterations we observe in the present investigation they will require further study.

## 6. EXERCISE-INDUCED SIGNAL TRANSDUCTION IS ALTERED IN TYPE II DIABETIC MUSCLE

A major finding of this investigation is that the phosphorylation (activation) of MAPK proteins in response to a maximal contractile stimulus appears to be altered in diabetic muscle. In addition, our data suggest that diabetes may alter this response differently in different muscle types. For example, in the lean plantaris, Erk1/2 activation typically did not reach maximal levels until three hours after cessation of exercise. Conversely, in the obese plantaris, Erk1/2 activation was maximal immediately following exercise and remained high through three hours post exercise (see Figure 2). Interestingly, this response appeared to be reversed in the lean and obese solei (see Figure 2). Similar to what we observed for the Erk1/2-MAPK proteins, the contraction-induced phosphorylation of p38- and Jnk-MAPK appeared to differ between the lean and obese animals. Unlike the regulation of the Erk1/2-MAPK proteins, differences between groups in the phosphorylation response of p38- and Jnk-MAPK appeared to manifest themselves during the later stages of the recovery period. For example, while contraction failed to increase p38*α* phosphorylation in the lean plantaris, p38*α* phosphorylation levels in the obese plantaris muscles were dramatically increased at the three hour post exercise time point (see Figure 3). Similarly, the degree of Jnk phosphorylation was significantly elevated in the obese, compared to lean plantaris muscles at the one hour post exercise time point (see Figure 4). To our knowledge, these findings have not been previously reported.

The time course and magnitude of increased MAPK phosphorylation following muscle contraction we observe in the lean animals are similar to that which have been reported previously following various modes of exercise in animal models as well as humans [45–51]. These responses appear to be physiologically relevant, as they were associated with the exercise-induced activation of p90RSK, a known downstream target of Erk1/2 [21]. Similar to what was observed with Erk1/2, the basal and contraction-induced phosphorylation levels of p90RSK were significantly higher in the obese, compared to lean plantaris at all time points. How Erk1/2 and p90RSK may function in the diabetic muscle or in producing exercise-induced muscle adaptation is not well understood. The p90RSK has been proven to be a ubiquitous and versatile mediator of Erk 1/2 signal transduction with the activation of p90RSK being thought to be involved in the regulation of nuclear factor-*κ*B and in the phosphorylation of transcription factors, including c-Fos, Nur77, and cAMP response element-bindingprotein [52]. The physiological significance of the apparent enhancement in Erk1/2 signaling we observe in diabetic muscle awaits further clarification. Nonetheless, in the light of previous data suggesting that MAPK and MAPK-associated signaling may be important upstream regulators of load-induced intracellular signaling events the results of the present study are consistent with previous work suggesting that diabetes may be associated by alterations in the ability of skeletal muscle to adapt to an exercise stimulus [7, 9, 53–56]. The extent to which the combined changes in tissue content and action of the Erk 1/2 proteins may play a role in the contracting diabetic muscle cannot be accurately assessed without further studies designed to evaluate the experimental manipulation of these proteins. Future studies employing methods to directly test the effects of altered Erk1/2 levels will with no doubt add to our understanding.

Why diabetes might be associated with differences in the contraction-induced activation of MAPK proteins is not readily apparent. It is thought that the MAPK proteins may play a key role in regulating muscle glucose uptake, and it has been hypothesized that the MAPK pathway may be a potential target for therapeutic strategies to restore the metabolic balance to type II diabetic patients. The differences in MAPK activation between normal and diabetic muscles we observed in the present study may be a compensatory mechanism to bypass defects in insulin dependent signaling [57]. Given the plethora of factors thought to be involved in regulating MAPK activation (e.g., mechanical stretch, muscle hypoxia, reactive oxygen levels, muscle damage, local release of growth factors, and cytokines, etc.) it is possible that the differences we observe between models could be due to several factors [24]. Because we examined the regulation of MAPK signaling following a series of in vivo contractions as opposed to using an in vitro experimental preparation it is unclear whether the alterations we found between models are a by product of differences in systemic environment of the exercising muscle or due to intrinsic alterations to the muscles themselves. Further research, perhaps using other experimental designs, will be with no doubt added to our understanding.

In conclusion, the results of this study have shown that contraction-induced regulation of MAPK signaling may be altered in the skeletal muscles of the obese Zucker rat. Because this model exhibits many of the same physiological characteristics seen in type II diabetic humans, these data suggest that diabetes may be associated with differences in how skeletal muscle “sense” and “respond” to a contractile stimulus. Whether similar findings would be observed in other types of diabetes (e.g., type I) or in humans remains to be determined. Given the potential of resistance training to improve the diabetic condition and the importance of MAPK signaling in regulating muscle adaptation, the findings of the current study may help to reveal new approaches for treating metabolic diseases and have applicability for the prescription of exercise programs aimed at improving muscle function in diabetic individuals.

## Figures and Tables

**Table 1:** Body weight and muscle mass of lean and obese (fa/fa) Zucker rats. An asterisk (*) indicates significant differences (*P* < .05) from the lean Zucker value.

	Lean Zucker	Obese Zucker
Body mass, g	328 ± 12.2	597 ± 21.7*
PLA mass, mg	373 ± .009	244 ± .007*
PLA mass/Body Wt (mg/g)	1.14 ± .43	0.41 ± .12*
SOL mass, mg	143 ± .005	146 ± .008
SOL mass/Body Wt (mg/g)	0.44 ± .13	0.24 ± .12*

## ABBREVIATIONS

MAPK: Mitogen-activated protein kinase
Erk1/2: Extracellular-signal-regulated kinase
Jnk: Jun N-terminus kinase
MAPKAPK-K2: MAPK-activated protein kinase-2
